# The role of foreign direct investments, urbanization, productivity, and energy consumption in Finland’s carbon emissions: an ARDL approach

**DOI:** 10.1007/s11356-023-28680-w

**Published:** 2023-07-10

**Authors:** Irina Georgescu, Jani Kinnunen

**Affiliations:** 1 Bucharest University of Economics, Calea Dorobanți, 15-17, Sector 1, 010552 București, Romania; 2 Åbo Akademi University, Tuomiokirkontori 3, 20500 Turku, Finland

**Keywords:** ARDL estimation, Productivity, Bounds test, Carbon dioxide emissions, Foreign direct investments, C32, C53, Q53

## Abstract

This study investigates the effects of productivity, energy consumption, foreign direct investments, and urbanization on carbon dioxide emissions (CO_2_) in Finland during 2000–2020 using an autoregressive distributed lag (ARDL) model. The results show that (i) there is evidence of cointegration among variables; (ii) energy consumption has a positive effect on CO_2_ emissions in the long run; (iii) labor productivity and urbanization have a negative effect on CO_2_ emissions in the long run; (iv) foreign direct investments are not a significant explainer of CO_2_ emissions. The results are discussed with some policy implications and suggested future research.

## Introduction

The relation between environmental pollution and economic growth has been a central topic of many studies (e.g., Adebayo and Beton Kalmaz 2021; Kartal 2022; 2023). Most part of CO_2_ emissions come from fossil fuel combustion. Economic growth, in particular economic activity, has as consequence the combustion of fossil fuels. CO_2_ emissions resulted mainly from fossil fuel consumption are the main source for environmental degradation. Non-renewable sources for energy use, mainly fossil fuels, generate greenhouse gases (GHG) which contribute to global warming. CO_2_ is the top GHG, followed by methane (CH_4_), nitrous oxides (N_2_O), and florinates gases. The present climate crisis starts from a great amount of CO_2_ emissions. The accelerated economic growth is associated with higher energy consumption and more CO_2_ emissions (Niyonzima et al. 2022).

This study investigates the determinants of CO_2_ emissions in Finland during 2000–2020. According to the Annual Climate Report 2021, Finland’s CO_2_ emissions declined by 9% from the previous year, and until 2035, Finland aims to be carbon neutral. The transition to a 2035 carbon neutrality economy could be done by low-carbon roadmaps specific to different sectors of industry (Majava et al. 2022). Another objective is to become the first fossil-free country in the world. Carbon neutrality can be achieved by balancing GHG emissions and sinks that sequester carbon. In Finland, the GHG emissions are generated from electricity consumption and industrial sectors such as metal, pulp and paper, and chemical and construction industries. Even if Nordic countries generate more renewable energy, they import carbon-based products. Nordic countries have the highest per capita electricity consumption, and 87% of their electricity generation is carbon-free. The Nordic electricity market is one of the most efficient, with a common share of renewables, an increased supply of electricity, and lower costs.

FDI inflows stimulate economic growth and factor productivity, by new investments, new jobs, and new technologies. Economic development and enhanced productivity require more energy consumption, faster urbanization, and increased pollutant emissions (Adebayo and Beton Kalmaz 2021). The purpose of the research supported by these remarks is to study the effect of FDI, productivity (PROD), energy consumption (ENCON), and urbanization (URB) on CO_2_ emissions for Finland. PROD as an independent variable is in a key role of economic growth as the growth in gross domestic product (GDP) per capita is broken down into growth in (labor) productivity growth (GDP/worked hours) and changes in labor utilization (worked hours per capita).

To study the determinants of CO_2_ emissions in Finland requires a complex approach which regards various sectors and encourages sustainable practices. The targets and policies to reduce CO_2_ emissions should cover multiple sectors, such as energy, transportation, industry, and agriculture. The use of renewable energy sources such as wind, solar, hydro, and biomass should be promoted. Enhancing energy efficiency and promoting sustainable transportation, agriculture and forestry, and foster circular economy principles require a collective effort involving government, businesses, and individuals. By implementing these strategies and fostering a culture of sustainability, Finland can make a significant progress in reducing its carbon footprint.

Kartal et al. (2022) focus on the influence of several indicators such as political stability, renewable energy, economic growth, and trade openness on Finland’s CO_2_ emissions applying non-linear and Fourier methods. Political stability appears as an important factor in achieving Finland’s goal of carbon neutrality by 2035. Kirikkaleli et al. (2023a) focus on the relation between environmental innovation and environmental sustainability in Norway, from 1990 to 2019 by a nonlinear ARDL model. Some results show that environmental innovation impacts positively the environment in Norway in the long term. Kirikkaleli et al. (2023b) conclude that in Sweden, energy productivity improves the environment by reducing CO_2_ emissions.

In the literature on the nexus between productivity and CO_2_ emissions, there are several recent works on this topic. Kirikkaleli and Ali (2023) find by Fourier-based estimators that environmental technology in Iceland improves the environment quality. Environmental technologies together with trade openness in Iceland contribute to the decrease of CO_2_ emissions generated by production. Applying a nonlinear ARDL approach for Sweden for the period 1980–2018, Adebayo et al. (2022) obtain that positive shocks in environmental technologies enhance air quality, decreasing CO_2_ emissions. The study by Kirikkaleli and Sowah (2023) reveals that for Finland, a change in energy productivity leads to a decrease in CO_2_ emissions, while economic growth and trade openness lead to an increase in CO_2_ emissions.

This study represents a contribution to the literature, by tackling a gap in the study of the relation between productivity and environmental damage for Finland as a Nordic country. New technologies enhance labor productivity, which contributes further to environmental pollution. Urbanization and globalization, as complex phenomena, facilitate investments through FDI. Globalization increases labor productivity, deteriorating air quality by increased industrialization, natural resource exploitation, displacement of local industries, and unequal distribution of environmental impacts. Therefore, this research arises from the interest to study these issues.

The work is organized as follows. The next section consists of a literature review and a research gap. The methodology section proposes the ARDL technique to investigate the impact of PROD, URB, ENCON, and FDI on CO_2_ emissions. The empirical results are followed by discussions and interpretations. The results show that in the long run, PROD and URB have negative effects on CO_2_ emissions, while ENCON has a positive effect. The squared productivity PROD2 proves to be not statistically significant. It follows that the EKC hypothesis does not hold for this model. The short-run effects of the dependent variable are mixed. The error correction term (ECT) is − 1.50, meaning that the speed of adjustment from the long-run to the short-run equilibrium is 150%. Conclusions and policy recommendations end the study. These results contribute significantly to the existing literature on productivity and environmental degradation and have implications for policy development in this field.

## Literature review

Several studies discussed the relation between air pollution and economic growth for Nordic countries. Alonso-Rodriguez (2017) proves that CO_2_ emissions in Finland affect those in Norway and Sweden, during 1960–2014, by means of a VAR(2) model and Granger causality. Maalej and Cabagnols (2022) apply ARDL for studying the correlation among renewable energy, economic activity, and technology in Denmark, Germany, and Finland. They concluded that gross fixed capital formation per capita affects positively Germany and Denmark’s GDP per capita, but for Finland, this effect exists only in the short run. Moreover, energy use impacts CO_2_ emissions positively in Germany and Denmark, but not Finland, because of the significant share of renewable energy in this country. In a study on Finland which includes quarterly data from 1990 to 2019, Kartal et al. (2022) analyze the effects of political stability on consumption-based carbon dioxide (CCO_2_) using non-linear and Fourier-based approaches. The main findings of this study are that positive shocks in economic growth have an increasing impact on CCO_2_, positive and negative shocks in renewable energy consumption have a decreasing impact on CCO_2_, and positive shocks in trade openness have a decreasing impact on CCO_2_. Overall, the study emphasizes the significant role of political stability in influencing CCO_2_. By promoting political stability and addressing positive shocks in economic growth, increasing renewable energy consumption and trade openness, policymakers can effectively work towards reducing CCO_2_ emissions and achieving their environmental goals. Using monthly data during 1973–2019, Kartal (2023) conducts an analysis for USA on the impact of energy consumption on CO_2_ emissions by dynamic ARDL (DYNARDL) simulations. These simulations proved that in the long run, fossils and nuclear energy have a positive effect on CO_2_ emissions, while renewable energy has a negative effect. Kartal (2022) explores the influence of energy consumption and various energy sources, including fossils fuels and nuclear and renewable energy on CO_2_ emissions focusing on the top five countries responsible for over 50% of global CO_2_ emissions. The empirical analysis is conducted using multivariate adaptive regression splines (MARS technique). Some results of the study are that the effects on CO_2_ emissions vary across the examined countries, but coal, oil, and natural gas are important determinants of CO_2_ emissions. The study sheds a light on the detrimental impact of fossil fuel usage on CO_2_ emissions, especially in high-carbon–emitting countries. Kartal et al. (2023) aim to assess the impact of energy consumption changes on CO_2_ emissions in France, employing DYNARDL. It follows that nuclear power has a negative effect on CO_2_ emissions, while renewable energy does not present a significant effect.

The environmental consequence of economic activity has been modeled by environmental Kuznets curve (EKC). EKC is an inverted U-shaped curve introduced by Grossman and Krueger (1991). According to EKC, at lower levels of income, environmental pollution increases; after a certain level of GDP per capita, pollution declines, and the economic development contributes to the increase of environmental quality. EKC is named after Kuznets (1955) who asserted that income inequality rises, and then diminishes when economic growth increases. By Rashid Gill et al. (2018), the EKC analysis is sensitive and depends on the form of the model.

The effects of labor productivity on CO_2_ emissions have been less extensively studied. Fitzgerald et al. (2018) and Chen et al. (2021) study the trade-off of CO_2_ emissions per capita and labor productivity based on EKC hypothesis and a fixed effects model in 36 OECD countries and China during the period 1990–2018. Their results confirm the N-shaped EKC suggesting that at the early development stage, CO_2_ emissions increase with labor productivity (positive relation), and after a certain threshold, CO_2_ emissions decrease with rising labor productivity (negative relation), and later, the CO_2_ emissions return to increasing trend with higher labor productivity. Amri et al. (2019) use total factor productivity as an indicator of economic growth and find that the EKC hypothesis for Tunisia is rejected.

Zhong and Su (2021) study labor market dynamics, labor productivity, and CO_2_ emissions in global value chains consisted of 44 economies. In their proposed approach, countries specialize according to their comparative advantages and stage of economic development. They argue for policy recommendations to develop the low-carbon technological know-how and apply such technologies through value chains. They call for global coordination of production networks and labor markets for better energy/climate planning. For reducing CO_2_ emissions, they argue that policies towards energy efficiency are the most important, and interestingly, that labor productivity improvement accounts the most for the growth of CO_2_ emissions. Fitzgerald et al. (2018) study the United States’ state-level CO_2_ emissions and average working hours, finding a strong positive relationship over different political, economic, and demographic drivers of CO_2_ emissions. They suggest that working time reduction can lower unemployment and enhance life quality, while reducing CO_2_ emissions.

Some works find a negative relation between environmental degradation and economic growth (Azam et al. 2016; Dogan and Aslan 2017); other works find it positive (Khan et al. 2019). A negative relation between CO_2_ emissions and economic growth can be explained by the application of efficient environmental policies.

Some studies validate the existence of an EKC (Kim 2019; Rashid Gill et al. 2019; Ali et al. 2021); others do not find any evidence (Magazzino 2014; 2015).

Androniceanu and Georgescu (2023) analyze a panel of 25 EU member states and discover by ARDL technique the positive impact of FDI, CO_2_ emissions, and energy consumption on economic growth by the existence of both long-run and short-run causality.

Muhammad (2019) analyzes the reciprocal effects among CO_2_ emissions, economic growth, and energy consumption for 68 developed, emerging, and MENA countries during 2001–2017. The results revealed a positive relation between economic growth and energy consumption for developed and emerging economies and a negative relation for MENA countries. Muhammad and Khan (2019) examine the effect of FDI, CO_2_ emissions, and energy consumption on economic growth for 34 host countries of Asia and 115 source countries during 2001–2012. The results demonstrate a positive relation between FDI and economic growth and energy consumption and economic growth respectively.

Bakhsh et al. (2021) analyze the linkage between FDI inflows and CO_2_ emissions for a panel of 40 Asian countries during 1996–2016 by means of generalized method of moments (GMM). FDI inflows in a host economy bring a transfer of new technologies and generate employment and economic development. This accelerated growth leads to a raised level of CO_2_ emissions. This explains the positive relation between FDI inflows and CO_2_ emissions found in Bakhsh et al. (2021). The negative correlation between FDI and CO_2_ might be caused by the financial system of the host economy or its capacity to absorb new technologies (Simionescu et al. 2017).

The relation between urbanization and air pollution is another significant research theme (de Leon Barido and Marshall 2014; Wang et al. 2021; Liu et al. 2022). Liu et al. (2022) discover that for China, urbanization has both positive and negative effects on air quality. Using fully modified ordinary least squares model (FMOLS), Liu et al. (2022) find that until 2013, this correlation was negative, and then it became positive. In the beginning, highly agglomerated cities, with powerful industries and urban congestion caused the generation of air pollutants, mainly particulate matter (PM_2.5_). By the effective application of environmental policies, urbanization policies have become low-carbon, and cleaner energy sources prevail. De Leon Barido and Marshall (2014) prove, by fixed and random effects for 80 countries during 1983–2005, that in higher-income countries, urbanization reduces pollutant emissions, while in lower-income countries, it increases them. The difference between the two categories of countries is that in higher-income countries, the income is differently generated and distributed. Moreover, higher-income countries are characterized by low income inequality and a higher potential to develop the infrastructure.

## Data and methodology

The main research variables and their sources are shown in Table 1. The study examines the long- and short-run effects of PROD and squared productivity PROD2, ENCON, FDI, and URB on CO_2_ emissions in Finland during 2000–2020.

**Table 1:** Variables and sources

Variable	Acronym	Measurement unit	Source
Annual CO_2_ emissions (per capita)	CO_2_	tons	Our World in Data (2000–2020)
Labor productivity growth	PROD	Annual growth rate (%)	OECD (2000–2020)
Final energy consumption	ENCON	Million tons of oil equivalent	Eurostat (2000–2020)
Foreign direct investments net inflows % of GDP	FDI	%	World Bank (2000–2020)
Urbanization	URB	%	World Bank (2000–2020)

Source: Author collection from various databases

Labor productivity growth means GDP per hour worked growth.

Non-stationary data are used to model the long-run equilibrium. Several cointegration techniques have been proposed by Engle and Granger (1987), Phillips and Hansen (1990), and Johansen (1988). For this study, the ARDL approach was chosen, a method which gained interest in the recent years by the works of Pesaran and Shin (1998) and Pesaran et al. (2001). The ARDL method tolerates different orders of integration I(0) and I(1) in the variables, or mutually cointegrated variables (Frimpong and Oteng 2006), being a more flexible method for econometric analysis. Moreover, the ARDL method provides unbiased estimates, not taking into account regressors’ endogeneity (Harris and Sollis 2003). The error correction model (ECM) integrates the short-run dynamics and the long-run equilibrium by means of lagged variables (Menegaki 2019).

The model specification is:

1 $${CO}_{2t}={a}_{0}+{a}_{1}{PROD}_{t}+{a}_{2}{PROD}_{t}^{2}+{a}_{3}{ENCON}_{t}+{a}_{4}{FDI}_{t}+{a}_{5}{URB}_{t}+{\varepsilon }_{t}$$

Equation (1) can be expressed as an ARDL regression:

2 $${\Delta CO}_{2t}={a}_{0}+\sum_{k=1}^{n}{a}_{1}{\Delta PROD}_{t-k}+\sum_{k=1}^{n}{a}_{2}\Delta {PROD}_{t-k}^{2}+\sum_{k=1}^{n}{a}_{3}{\Delta ENCON}_{t-k}+\sum_{k=1}^{n}{a}_{4}{\Delta FDI}_{t-k}+\sum_{k=1}^{n}{a}_{5}{\Delta URB}_{t-k}+{\lambda }_{1}{PROD}_{t-1}+{\lambda }_{2}{PROD}_{t-1}^{2}+{\lambda }_{3}{ENCON}_{t-1}+{\lambda }_{4}{FDI}_{t-1}+{\lambda }_{5}{URB}_{t-1}+{\varepsilon }_{t}$$

In Eq. (2), ${a}_{0}$ is the drift component, $\Delta$ is the first difference, and ${\varepsilon }_{t}$ is the white noise. The long-run causality between variables exists; therefore, ECM has the form:

3 $${\Delta CO}_{2t}={a}_{0}+\sum_{k=1}^{n}{a}_{1}{\Delta PROD}_{t-k}+\sum_{k=1}^{n}{a}_{2}\Delta {PROD}_{t-k}^{2}+\sum_{k=1}^{n}{a}_{3}{\Delta ENCON}_{t-k}+\sum_{k=1}^{n}{a}_{4}{\Delta FDI}_{t-k}+\sum_{k=1}^{n}{a}_{5}{\Delta URB}_{t-k}+\Gamma {ECM}_{t-1}+{\varepsilon }_{t}$$

In Eq. (3), $\Gamma$ is the coefficient of the ECM for the short-run dynamics.

The robustness of ARDL-ECM model is checked by means of serial correlation test, heteroskedasticity test, and Jarque Bera normality test.

The stability of the model is checked by the cumulative sum (CUSUM) and cumulative sum of square (CUSUMSQ) tests (Brown et al. 1975). Works by Pesaran and Shin (1998) and Pesaran et al. (2001) assert that the two tests reveal the fitness of the ARDL-ECM model. The two tests plot the residuals of ECM. If the plots of CUSUM and CUSUM of squares are within the 5% critical bound, then one cannot reject the null hypothesis of the stability of the parameters.

## Empirical results and discussion

**Fig. 1:**
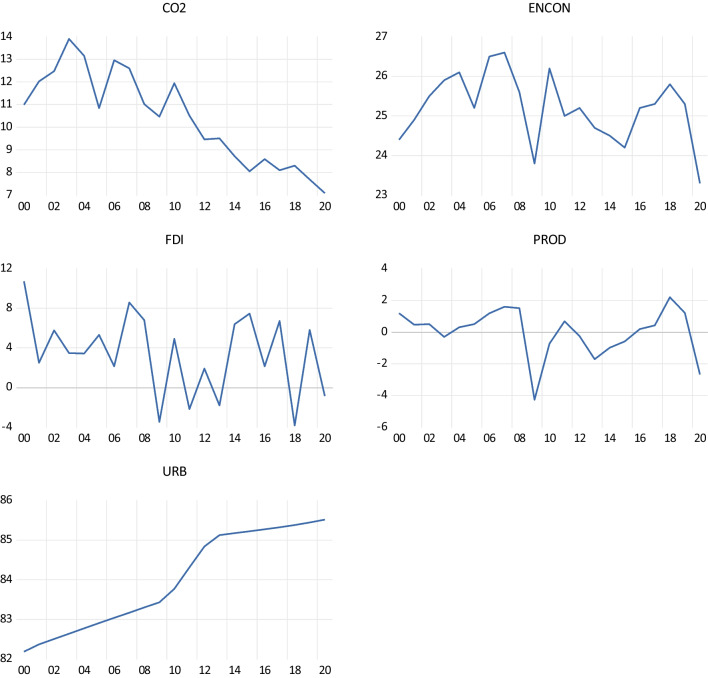
The evolution of CO_2_, ENCON, FDI, PROD and URB for Finland (2000–2020). Source: author calculation

Figure 1 shows the evolution of the five indicators for Finland during 2000–2020. One can see an increasing evolution of URB in Finland, especially during 2012–2020. Also, one notices a continuing decrease in CO_2_ emissions in Finland starting from 2010. Starting from 2018, ENCON abruptly declined, concurrently with PROD. During the 21-year research period, CO_2_ emissions in Finland were trending downwards since 2003, while urbanization increased every year; productivity, even relatively low in Finland compared to its competitors, increased in 14 years; energy consumption decreased during 9 years. Energy consumption and productivity declined in 2007–2009 as well as 2018 onwards.

Table 2 reports descriptive statistics for the studied variables. The mean of CO_2_ emissions is 10.40, with 7.09 and 13.9 being the lowest and highest values. The standard deviation of CO_2_ emissions is 2.03, indicating low variability. The average value for PROD is 0.01 and for ENCON is 25.2 with a low variability of 0.85. Kurtosis shows that ENCON, CO_2_, FDI, and URB have platykurtic distributions, while PROD has a leptokurtic distribution. ENCON, FDI, URB, and PROD have negative-skewed distributions, meaning that their left tails are longer, and the mass of the distribution is located on the right. CO_2_ has a positive-skewed distribution, and thus, its right tail is longer, and the mass of the distribution is located on the left.

**Table 2:** Descriptive statistics

	CO_2_	FDI	URB	PROD	ENCON
Mean	10.40	3.41	83.98	0.01	25.2
Median	10.51	3.47	83.77	0.41	25.2
Maximum	13.90	10.71	85.51	2.19	26.6
Minimum	7.09	− 3.83	82.18	− 4.27	23.3
Std. Dev	2.03	4.04	1.22	1.51	0.85
Skewness	0.03	− 0.30	− 0.02	− 1.21	− 0.35
Kurtosis	1.76	2.21	1.34	4.42	2.67
Jarque–Bera	1.33	0.85	2.40	6.98	0.52
Probability	0.51	0.65	0.29	0.030	0.76

Source: author calculation

First, some conventional tests are performed to check the data stationarity at level and at first difference: augmented Dickey-Fuller (ADF) test (Dickey and Fuller 1979). All variables must be stationary at level I(0) or at first difference I(1) in order to apply ARDL bounds test. The use of non-stationary data may lead to spurious regression. One can see that none of the series is integrated of order two. Therefore, ARDL technique is the most appropriate model, because it is not biased, and it is superior to other small sample size models.

The null hypothesis of ADF unit root tests assumes the presence of the unit root, while the alternative hypothesis is the absence of the unit root and that the time series is stationary. It follows from Table 3 that PROD, PROD2, ENCON, and FDI are I(0), while CO_2_ and URB are I(1). In Table 3, the probabilities are shown in parentheses.

**Table 3:** ADF unit root test

Variables	Level	First difference	Order of integration
	*T*-statistics	*T*-statistics	
CO_2_	0.24 (0.967)	− 5.40 (0.000)	I(1)
PROD	− 3.46 (0.020)	− 4.22 (0.004)	I(0)
PROD2	− 4.10 (0.005)	− 7.15 (0.000)	I(0)
ENCON	− 3.05 (0.046)	− 5.23 (0.000)	I(0)
FDI	− 6.11 (0.000)	− 10.35 (0.000)	I(0)
URB	− 0.98 (0.732)	− 3.32 (0.028)	I(1)

Source: author calculation

The optimal lag number of the vector autoregression (VAR) model is 2, as indicated by all criteria in Table 4.

**Table 4:** VAR Lag order selection criteria

Lag	LogL	LR	FPE	AIC	SC	HQ
0	− 178.83	NA	11.35	19.45	19.75	19.50
1	− 110.26	86.62	0.45	16.02	18.11	16.38
2	− 22.84	55.21*	0.01*	10.61*	14.49*	11.27*

Source: author calculation

*LR* sequential modified LR test statistic (each test at 5% level), *FPE* final prediction error, *AIC* Akaike information criterion, *SC* Schwarz information criterion, *HQ* Hannan-Quinn information criterion

*Lag order selected by the criterion

Next, the ARDL model will be applied to examine the dependence between CO_2_ and the independent variables PROD, PROD2, ENCON, FDI, and URB. The first step to be taken before estimating an ARDL model is to check the cointegration, and this is done using the bound tests, which involves rejecting or accepting the null hypothesis which says that the variables are not cointegrated.

Based on AIC lag criterion, the selected model is ARDL(2,2,1,2,2,2). The results of cointegration bounds test are presented in Table 5.

**Table 5:** Results of cointegration bounds test

Test statistic	Value	*K* (number of regressors)
F-statistic	7.15	5
Critical value bounds		
Significance	I(0)	I(1)
10%	2.33	3.41
5%	2.80	4.01
1%	3.9	5.41

Source: author calculation

Since F-calculated is 7.15, greater than the critical upper bound denoted by I(1), one considers that there is evidence of cointegration among variables. The estimated long-run coefficients are shown in Table 6.

**Table 6:** The long-run estimated coefficients

Variables	Coefficient	*T*-statistics	Prob
PROD	− 0.55	− 5.23	0.034
PROD2	0.10	0.23	0.833
FDI	− 0.03	− 0.65	0.577
URB	− 1.39	− 14.62	0.004
ENCON	1.23	5.64	0.029
C	96.81	7.42	0.017

Source: author calculation

From Table 6, one can see that PROD has a negative and statistically significant influence on CO_2_ at 5% level of significance. A 1% increase in PROD exerts a 0.55% decrease in CO_2_. Increased productivity is a driver to economic growth, which leads to a higher income and less CO_2_ emissions; hypothesis confirmed by Narayan et al. (2016). The negative effect of productivity on CO_2_ emissions is a new finding in the context of CO_2_ determinants. It suggests that the achieved economic expansion due to increased productivity may be achieved by cleaner low-carbon production. The effect of PROD2 on CO_2_ is not statistically significant, which means that the EKC hypothesis is rejected for this model.

FDI has a negative and not statistically significant influence on CO_2_. Also, there is 1.39% decrease in CO_2_ for a one-unit growth in URB. This negative relation between URB and CO_2_ emissions is confirmed by Muñoz et al. (2020) and Zhang et al. (2020). One reason for this negative relation would be the economy of scale effect of urbanization which contributes to carbon emissions reduction.

Table 6 reveals that ENCON exerts a positive impact on Finland’s CO_2_ emissions in the long run. A 1% increase in ENCON increases CO_2_ emissions by 1.23%. The economic implication of this result is that ENCON is the main factor which causes CO_2_ emissions, even if Finland is one of the countries which have an important share of renewable energy. This positive relation is also obtained by Tong et al. (2020) for a panel of E7 countries for period 1990–2014 using a bootstrap ARDL bound test.

The results of ARDL-ECM model are captured in Table 7. ECT is − 1.50, negative, and statistically significant at 5% level of significance, showing that there is evidence of cointegration. The speed of adjustment to long-run equilibrium after a deviation has occurred in the short run is 150%. The adjustment coefficient of − 1.50 indicates that the deviations of CO_2_ from equilibrium are corrected by 150% in the next period. The short-run dynamics of PROD, FDI, URB, and ENCON are mixed.

**Table 7:** Short-run ARDL approach

Variable	Coefficient	*T*-statistics	Prob
D(CO_2_(-1))	0.61	7.62	0.016
D(PROD)	− 0.62	− 18.47	0.002
D(PROD(-1))	0.42	8.59	0.013
D(PROD2)	− 0.06	− 3.98	0.014
D(FDI)	− 0.03	− 3.08	0.091
D(FDI(-1))	0.07	8.38	0.013
D(URB)	− 4.76	− 9.48	0.010
D(URB(-1))	4.20	8.16	0.014
D(ENCON)	1.58	27.62	0.001
D(ENCON(-1))	− 0.57	− 4.97	0.038
CointEq(-1)	− 1.50	− 14.15	0.005
R-squared	0.99		
Adjusted R-squared	0.98		

Source: author calculation

In this case, ECT is between − 2 and − 1, causing dampening oscillations. This means that the error correction process varies around the long-run value in a dampening approach (Too et al. 2021). The regressors jointly explain 98% of the total variation in CO_2_, as shown by adjusted R-squared.

### Diagnostic and stability tests

Table 8 contains the null hypothesis *H*_0_ of four diagnostic tests and their values. The *p*-values of serial correlation test, heteroskedasticity test, and Jarque Bera normality test are greater than 0.05, which is desirable. So, this model does not have autocorrelation and heteroskedasticity. The probability of Jarque–Bera test being greater than 0.05 and the Jarque–Bera value being less than 5, it follows that the residuals are normally distributed (Teyyare 2018). The Ramsey RESET test shows that the model is correctly specified; therefore, there is no instability in the determinants of CO_2_ emissions.

**Table 8:** Results of diagnostic and stability tests

Test	*H*_0_	Decision Statistics (*p*-value)
SC*	There is no serial correlation in the residuals	Accept *H*_0_ 81.75 [0.070]
HE**	There is no autoregressive conditional heteroskedasticity	Accept *H*_0_ 0.15 [0.990]
NO***	Normal distribution	Accept *H*_0_ 2.63 [0.268]
RR***	Absence of model misspecification	Accept *H*_0_ 1.39 [0.396]

Source: author calculation

SC* Serial correlation, **Heteroscedasticity, ***Normal Distribution, ***Ramsey RESET

**Fig. 2:**
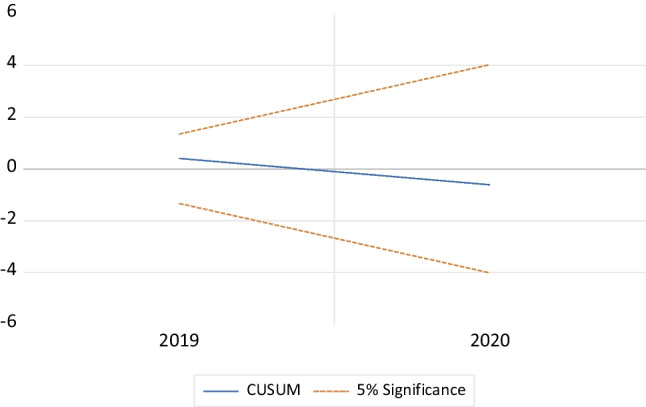
Plot of CUSUM for coefficients’ stability of ARDL model at 5% level of significance. Source: author calculation

**Fig. 3:**
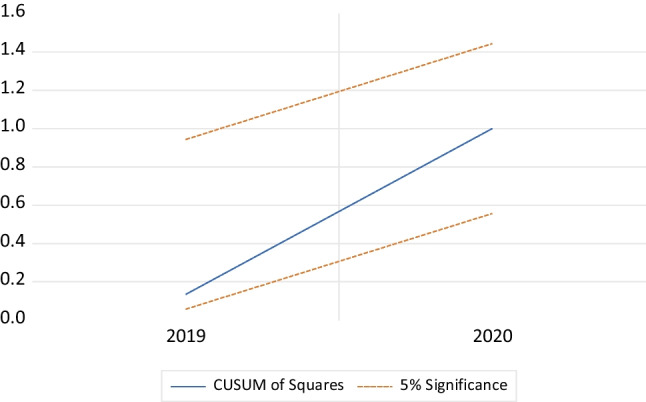
Plot of CUSUMSQ for coefficients’ stability of ARDL model at 5% level of significance. Source: author calculation

The stability of the model is tested using the CUSUM and CUSUM of squares tests (Tanizaki 1995). In our model, both tests suggest that the parameters are stable, because the plots of CUSUM and CUSUM of squares lie within the 5% critical bound represented by the red dashed line. This points out to the model’s suitability to be used for predictions (Figs. 2 and 3).

The post estimation tests of the ARDL-ECM model confirm that the dynamic properties of the time series are ensured. Therefore, based on the robustness checks, ARDL-ECM has a good fit; therefore, policy recommendations may be suggested.

## Conclusions and recommendations

This study brings a contribution to the existing literature by taking into account the impact of foreign direct investments, urbanization, energy consumption, and labor productivity on CO_2_ emissions for Finland during 2000–2020. However, economic growth was replaced by the greatest productivity driver of growth, labor productivity.

The ARDL bounds test showed that the variables are cointegrated. In the long run, CO_2_ emissions are positively connected with energy consumption, and negatively connected with productivity and urbanization. The Nordic electricity system is one of the most efficient markets. The energy and climate policies are steady and rather efficient, and in the long term, they stimulate renewables, increase carbon taxes, and decarbonize energy supply. The EKC hypothesis does not hold for this model, indicating that other factors not included in the model may influence the environmental damage, or the relation between productivity and air pollution is more complex and not adequately modeled by a quadratic equation.

A problem in terms of Finland’s weak competitiveness is relatively low productivity, which suggests that policy efforts should be put on increasing labor productivity through automation and innovation, which could also lead to shorter work week (cf. Fitzgerald et al. 2018), and dismantling the collective labor agreements even for some other countries in a different position of a global value chain, increased labor productive can be seen problematic (cf. Zhong and Su 2021).

One limitation of the study is that the econometric analysis has been done aggregately, not by economic sectors. The effects of Covid 19 pandemic could be studied when recent data is available.

Renewable energy has a key role in addressing global warming and other environmental issues. A holistic approach is necessary to address climate change comprehensively, which included energy efficiency measures, sustainable land use practices, and changes in consumer patterns. These factors combined could accelerate the transition to a low-carbon future.

## Data Availability

The data can be available upon request.

## References

[CR1] Adebayo TS, Beton Kalmaz D (2021). Determinants of CO_2_ emissions: empirical evidence from Egypt. Environ Ecol Stat.

[CR2] Adebayo TS, Oladipupo SD, Kirikkaleli D (2022). Asymmetric nexus between technological innovation and environmental degradation in Sweden: an aggregated and disaggregated analysis. Environ Sci Pollut Res.

[CR3] Agrawal R, Wankhede VA, Kumar A, Upadhyay A, Garza-Reyes JA (2021). Nexus of circular economy and sustainable business performance in the era of digitalization. Int J Product Perform Manag.

[CR4] Air quality in Finland (2023) Available at: https://www.iqair.com/finland Accessed 2 July 2023

[CR5] Ali AN, Akter S, Fogarassy C (2021). The role of the key components of renewable energy (combustible renewables and waste) in the CO_2_ emissions and economic growth of selected countries in Europe. Energies.

[CR6] Alonso-Rodriguez A (2017) The CO_2_ emissions in Finland, Norway and Sweden: a dynamic relationship, EconStor Preprints 171259, ZBW - Leibniz Information Centre for Economics. 10.20944/preprints201711.0098.v1 . Available at:https://europepmc.org/article/PPR/PPR51781 . Accessed 2 July 2023

[CR7] Amri F, Zaied YB, Lahouel BB (2019). ICT, total factor productivity, and carbon dioxide emissions in Tunisia. Technol Forecast Soc Chang.

[CR8] Androniceanu A, Georgescu I (2023). The impact of CO_2_ emissions and energy consumption on economic growth: a panel data analysis. Energies.

[CR9] Azam M, Khan AQ, Abdullah HB, Qureshi ME (2016). The impact of CO_2_ emissions on economic growth: evidence from selected higher CO2 emissions economies. Environ Sci Pollut Res.

[CR10] Bakhsh S, Yin H, Shabir M (2021). Foreign investment and CO_2_ emissions: do technological innovation and institutional quality matter? Evidence from system GMM approach. Environ Sci Pollut Res.

[CR11] Brown RL, Durbin J, Evans JM (1975). Techniques for testing the constancy of regression relationships over time. J R Stat Soc Ser B (Methodological).

[CR12] Chen X, Ma W, Valdmanis V (2021). Can labor productivity growth reduce carbon emission? Evidence from OECD countries and China. Manag Environ Qual.

[CR13] de Leon Barido DP, Marshall JD (2014). Relationship between urbanization and CO2 emissions depends on income level and policy. Environ Sci Technol.

[CR14] Dickey D, Fuller W (1979). Distribution of the estimator for autoregressive time series with a unit root. J Am Stat Assoc.

[CR15] Dogan E, Aslan A (2017). Exploring the relationship among CO_2_ emissions, real GDP, energy consumption and tourism in the EU and candidate countries: evidence from panel models robust to heterogeneity and cross-sectional dependence. Renew Sustain Energy Rev.

[CR16] Engle RF, Granger CW (1987). Cointegration and error correction: representation, estimation and testing. Econometrica.

[CR17] Fitzgerald JB, Schor JB, Jorgenson AK (2018). Working hours and carbon dioxide emissions in the United States, 2007–2013. Soc Forces.

[CR18] Frimpong MJ, Oteng EF (2006). Bound testing approach: an examination of foreign direct investment, trade and growth relationships. MPRA Paper No.

[CR19] Grossman GM, Krueger AB (1991) Environmental impacts of a North American Free Trade Agreement. NBER Working Papers 3914. Accessed 2 July 2023

[CR20] Harris R, Sollis R (2003). Applied time series modelling and forecasting.

[CR21] Johansen S (1988). Statistical analysis of cointegration vectors. J Econ Dyn Control.

[CR22] Johansen S, Juselius K (1990). Maximum likelihood estimation and inference on cointegration with applications to the demand for money. Oxford Bull Econ Stat.

[CR23] Kartal MT (2022). The role of consumption of energy, fossil sources, nuclear energy, and renewable energy on environmental degradation in top-five carbon producing countries. Renew Energy.

[CR24] Kartal MT (2023). Production-based disaggregated analysis of energy consumption and CO2 emission nexus: evidence from the USA by novel dynamic ARDL simulation approach. Environ Sci Pollut Res.

[CR25] Kartal MT, Depren SK, Kirikkaleli D, Depren Ö, Khan U (2022). Asymmetric and long-run impact of political stability on consumption-based carbon dioxide emissions in Finland: evidence from nonlinear and Fourier-based approaches. J Environ Manag.

[CR26] Kartal MT, Pata UK, Depren SK, Depren Ö (2023). Effects of possible changes in natural gas, nuclear, and coal energy consumption on CO2 emissions: evidence from France under Russia’s gas supply cuts by dynamic ARDL simulations approach. Appl Energy.

[CR27] Khan I, Khan N, Yaqub A, Sabir M (2019). An empirical investigation of the determinants of CO_2_ emissions: evidence from Pakistan. Environ Sci Pollut Res.

[CR28] Kim S (2019). CO_2_ emissions, energy consumption, GDP and foreign direct investment in ANICs countries. Contemp Issues Appl Econ.

[CR29] Kirikkaleli D, Ali K (2023). Patents on environmental technologies and environmental degradation in a Scandinavian country: evidence from novel Fourier-based estimators. Geol J.

[CR30] Kirikkaleli D, Sowah JK (2023). The asymmetric and long run effect of energy productivity on quality of environment in Finland. J Clean Prod.

[CR31] Kirikkaleli D, Addai K, Karmoh JS (2023). Environmental innovation and environmental sustainability in a Nordic country: evidence from nonlinear approaches. Environ Sci Pollut Res.

[CR32] Kirikkaleli D, Sowah JK, Addai K, Altuntaş M (2023). Energy productivity and environmental quality in Sweden: evidence from Fourier and non-linear based approaches. Geol J.

[CR33] Kuznets S (1955). Economic growth and income inequality. Am Econ Rev.

[CR34] Liu H, Cui W, Zhang M (2022). Exploring the causal relationship between urbanization and air pollution: evidence from China. Sustain Cities Soc.

[CR35] Maalej A, Cabagnols A (2022) CO_2_ emissions and growth: exploring the nexus between renewable energies, economic activity and technology. J Energy Dev 46(1–2). Accessed 2 July 2023

[CR36] Magazzino C (2014). The relationship between CO_2_ emissions, energy consumption and economic growth in Italy. Int J Sustain Energ.

[CR37] Magazzino C (2015). Economic growth, CO_2_ emissions and energy use in Israel. Int J Sust Dev World.

[CR38] Majava A, Vadén T, Toivanen T, Järvensivu P, Lähde V, Eronen JT (2022). Sectoral low-carbon roadmaps and the role of forest biomass in Finland’s carbon neutrality 2035 target. Energy Strateg Rev.

[CR39] Menegaki AN (2019). The ARDL method in the energy-growth nexus field; best implementation strategies. Economies.

[CR40] Muhammad B (2019). Energy consumption, CO_2_ emissions and economic growth in developed, emerging and Middle East and North Africa countries. Energy.

[CR41] Muhammad B, Khan S (2019). Effect of bilateral FDI, energy consumption, CO_2_ emission and capital on economic growth of Asia countries. Energy Rep.

[CR42] Muñoz P, Zwick S, Mirzabaev A (2020). The impact of urbanization on Austria’s carbon footprint. J Clean Prod.

[CR43] Narayan PK, Saboori B, Soleymani A (2016). Economic growth and carbon emissions. Econ Model.

[CR44] Niyonzima P, Yao X, Ofori EK (2022). How do economic growth and the emissions of carbon dioxide relate?. Open Access Libr J.

[CR45] Parikh J, Shukla V (1995). Urbanization, energy use and greenhouse effects in economic development - results from a crossnational study of developing countries. Glob Environ Chang.

[CR46] Pesaran MH, Shin Y (1998). An autoregressive distributed-lag modelling approach to cointegration analysis. Econ Econ Theory 20th Century: Ragnar Frisch Centennial Symp.

[CR47] Pesaran MH, Shin Y, Smith RJ (2001). Bounds testing approaches to the analysis of level relationships. J Appl Econ.

[CR48] Phillips PCB, Hansen BE (1990). Statistical inference in instrumental variables regression with I(1) processes. Rev Econ Stud.

[CR49] Rashid Gill A, Viswanathan KK, Hassan S (2018). The environmental Kuznets curve (EKC) and the environmental problem of the day. Renew Sustain Energy Rev.

[CR50] Rashid Gill A, Hassan S, Viswanathan KK (2019). Is democracy enough to get early turn of the environmental Kuznets curve in ASEAN countries. Energy Environ.

[CR51] Simionescu M, Lazányi K, Sopková G, Dobeš K, Balcerzak AP (2017). Determinants of economic growth in V4 countries and Romania. J Compet.

[CR52] Tanizaki H (1995). Asymptotically exact confidence intervals of CUSUM and CUSUMSQ tests: a numerical derivation using simulation technique. Commun Stat-Simul Comput.

[CR53] Teyyare E (2018). An analysis of the savings-investment-corporate quality relationship: the case of Turkey. Bolu Abant Izzet Baysal University. J Soc Sci Inst.

[CR54] Tong T, Ortiz J, Xu C, Li F (2020). Economic growth, energy consumption, and carbon dioxide emissions in the E7 countries: a bootstrap ARDL bound test. Energy Sustain Soc.

[CR55] Too HK, Bett HK, Gitau R (2021). What explains the trends of wheat imports in Kenya; a cointegration analysis using ARDL-ECM modelling. J Econ Sustain Dev.

[CR56] Wang Y, Yao L, Xu Y, Sun S, Li T (2021). Potential heterogeneity in the relationship between urbanization and air pollution, from the perspective of urban agglomeration. J Clean Prod.

[CR57] Zhang X, Geng Y, Shao S, Wilson J, Song X, You W (2020). China’s non-fossil energy development and its 2030 CO2 reduction targets: the role of urbanization. Appl Energy.

[CR58] Zhong S, Su B (2021). Assessing the effects of labor market dynamics on CO2 emissions in global value chains. Sci Total Environ.

